# Characterization of the Active Ingredients and Prediction of the Potential Anticolitis Mechanism of the Feng-Liao-Chang-Wei-Kang Capsule via Mass Spectrometry and Network Pharmacology

**DOI:** 10.1155/jamc/2948965

**Published:** 2025-05-05

**Authors:** Tingting Liu, Zhijiang He, Witiao Lv, Liyun Deng, Xizhe Sun, Yanfei Chen

**Affiliations:** ^1^School of Hainan Provincial Drug Safety Evaluation Research Center, Hainan Medical University, Haikou, China; ^2^Department of Orthopedics, Hainan Provincial Corps Hospital of Chinese People's Armed Police Force, Haikou, China; ^3^School of Pharmacy, Hainan Medical University, Haikou, China

**Keywords:** anticolitis mechanism, characterization of active ingredients, Feng-Liao-Chang-Wei-Kang capsule, mass spectrometry, network pharmacology

## Abstract

The Feng-Liao-Chang-Wei-Kang (FLCWK) capsule is a nationally protected Chinese patent medicine for the treatment of colitis. However, the potential active components and the pharmacological mechanism underlying the anticolitis effect of the FLCWK capsule remain unclear. This study aimed to reveal the active ingredients and possible anticolitis mechanism of the FLCWK capsule using an integrated approach combining mass spectrometry and network pharmacology analysis. Ultra-performance liquid chromatography plus Q-Exactive Orbitrap tandem mass spectrometry (UPLC-Q-Exactive Orbitrap MS) was applied to identify the components of the FLCWK capsule. A network pharmacology study, including target gene prediction and functional enrichment, was applied to screen the active ingredients of the FLCWK capsule and explore its potential mechanism for the treatment of colitis. A total of 115 components were identified in the FLCWK capsule. Network pharmacology results showed that 46 of these compounds with good bioavailability and drug-likeness, such as 4′,5-dihydroxyflavone, pinostrobin, naringenin chalcone, apigenin, and morin, were selected as active ingredients. The active ingredients may act on 352 core protein targets, including EGFR, AKT1, PIK3R1, PIK3CB, and MAPK1, thereby modulating relevant pathways, such as MAPK and PI3K-Akt signaling pathways, and thus alleviating inflammation and intestinal damage in colitis. This study provided a useful approach to identify active components and the anticolitis mechanism of the FLCWK capsule and built up a reliable foundation for its clinical treatment.

## 1. Introduction

Colitis, an inflammatory bowel disease (IBD), is characterized by mucosal epithelial damage and disruption of intestinal homeostasis [1]. The clinical manifestations of colitis include bellyache, diarrhea, and bloody stool, which seriously compromise the life quality of patients [2]. Current therapeutic strategies primarily rely on aminosalicylic acid derivatives, immunosuppressive steroids, and biological agents [3]. However, the long-term administration of these medications is limited by drug resistance and toxicity, underscoring the need for safer and more effective complementary and adjuvant therapies [4]. Traditional Chinese herbal medicine has shown promise in colitis management, particularly the Feng-Liao-Chang-Wei-Kang (FLCWK) capsule, a nationally protected Chinese patent medicine (protection number: ZYB2072004057) [5–8]. The FLCWK capsule containing *Daphniphyllum calycinum* Benth. and *Polygonum hydropiper* Linn. has emerged as a particularly noteworthy therapeutic agent for colitis treatment [7, 8]. In clinical practice, the FLCWK capsule is commonly administered with mesalazine, with clinical studies demonstrating its capacity to enhance therapeutic outcomes [6, 9]. For example, a clinical study involving 120 patients with chronic colitis demonstrated that combination therapy with mesalazine and FLCWK significantly improved clinical outcomes compared to mesalazine monotherapy. The combination group exhibited superior clinical efficacy (*p* < 0.05), significantly reduced inflammatory markers (IL-6, CRP, TNF-α; *p* < 0.001), decreased mucosal lesions (*p* < 0.001), and enhanced quality of life (*p* < 0.001) [6]. Similarly, a study by Hou and Gan involving 52 patients with recurrent ulcerative colitis revealed that the combination of mesalazine and FLCWK resulted in a higher total effective rate (92.31% vs. 69.23%, *p* < 0.05) compared to mesalazine alone [9]. However, the potential active components and the pharmacological mechanism underlying the anticolitis effect of the FLCWK capsule remain unclear.

Orbitrap mass spectrometry (MS) is a powerful analytical platform for characterizing complex chemical compositions in Chinese herbal medicines, offering high sensitivity, resolution, and a broad dynamic range [10, 11]. Its ability to analyze MS^n^ fragments enables precise structural elucidation of compounds, making it ideal for investigating the chemical profile of the FLCWK capsule [12, 13]. Network pharmacology is well-suited for studying Chinese medicine due to its multicomponent, multitarget, and multipathway nature [10, 14–16]. It has been widely used to investigate interactions between herbal compounds and disease mechanisms, providing a robust framework for predicting the FLCWK capsule's active components and anticolitis mechanisms [17–19].

In conclusion, in this study, the chemical constituents of the FLCWK capsule were analyzed efficiently and accurately by using ultra-performance liquid chromatography plus Q-Exactive Orbitrap tandem mass spectrometry (UPLC-Q-Exactive Orbitrap MS). Then, network pharmacology was employed to illustrate the potential active components and anticolitis mechanisms of the FLCWK capsule. The obtained results could be helpful to build up reliable information on the clinical application of FLCWK capsules in the treatment of colitis.

## 2. Materials and Methods

### 2.1. Reagents and Materials

The FLCWK capsule (0.37 g per capsule, lot number: 221103) was produced by Haikou Qili Pharmacy Co., Ltd. (Haikou, China). The 99 reference compounds of the FLCWK capsule were purchased from Chemexpress Co., Ltd. (Shanghai, China) and Sigma-Aldrich (St. Louis, MO, USA). The purity of all standard compounds was determined to be higher than 98%. The name, molecular formula, batch number, and company of each reference compound are shown in Table S1. Acetonitrile, methanol, and formic acid were HPLC grade and purchased from Thermo Fisher Scientific (Fair Lawn, NJ, USA).

### 2.2. Analysis of the FLCWK Capsule Chemical Components

#### 2.2.1. Preparation of the Sample Solution

Precisely 0.4 g of FLCWK capsule contents were weighed and transferred into a 50-mL volumetric flask. Subsequently, 40 mL of 80% methanol was added, followed by ultrasonication for 20 min. After cooling to ambient temperature, the solution was brought to volume with 80% methanol and homogenized by thorough mixing. A 2-mL aliquot of this solution was then quantitatively transferred and diluted to 10 mL with 80% methanol. The diluted solution was filtered through a 0.22-μm microporous membrane. For final preparation, 1 mL of the filtrate was mixed with an equivalent volume of 80% methanol to obtain the test sample solution. The prepared sample (5 μL) was subsequently injected into the UPLC-Q-Exactive Orbitrap system for analysis.

#### 2.2.2. UPLC-Q-Exactive Orbitrap MS Conditions

UPLC analysis was carried out on an ACQUITY UPLC I-Class plus system (Waters Co., Milford, MA, USA). The separation was performed on an ACQUITY UPLC HSS T3 column (100 × 2.1 mm, 1.8 μm) maintaining a flow rate of 0.35 mL/min at 45°C with a 5-μL injection volume. The mobile phase consisted of A (water with 0.1% formic acid) and B (acetonitrile), with the following elution gradient program: 0.0 min A:B (95:5) ⟶ 2.0 min A:B (95:5) ⟶ 4.0 min A:B (70:30) ⟶ 8.0 min A:B (50:50) ⟶ 10.0 min A:B (20:80) ⟶ 14.0 min A:B (0:100) ⟶ 15.0 min A:B (0:100) ⟶ 15.1 min A: B (95:5) ⟶ 16.0 min A:B (95:5).

MS analysis was performed on a Q-Exactive Orbitrap MS (Thermo Fisher Scientific, Fair Lawn, NJ, USA) connected to an electrospray ionization (ESI) source operating in both positive and negative modes. The mass range was 100–1200 m/z, the nitrogen sheath gas flow rate was 35 Arb, the auxiliary gas was 8 Arb, the capillary temperature was 320°C, and the spray voltage in the positive and negative mode was 3800 and −3000 V, respectively.

#### 2.2.3. Compound Identification

The raw data were acquired using the Xcalibur 4.1 software (Thermo Fisher Scientific, Fair Lawn, NJ, USA), and all obtained data were processed by the Compound Discoverer (CD) 3.0 (Thermo Fisher Scientific, Fair Lawn, NJ, USA) and Xcalibur 4.1 software packages. The compounds were identified by comparing the chromatographic feature, empirical molecular formulas, and characteristic fragment ions with those of reference compounds or published known compounds in the HERB database (https://herb.ac.cn/).

### 2.3. Network Pharmacology

#### 2.3.1. Screening of Active Ingredients and Potential Targets of the FLCWK Capsule

The absorption, distribution, metabolism, excretion, and toxicity (ADMET) profiling of the identified components from the FLCWK capsule was estimated using the SwissADME online server (https://www.swissadme.ch/) [20]. The screening of the active ingredients was based on the following characteristics: (i) gastrointestinal absorption (GI absorption) was “High,” indicating good oral bioavailability and absorption of the ingredient; (ii) at least two of the five categories (Lipinski, Ghose, Veber, Egan, and Muegge) were set to “Yes”, indicating that the compound has good drug-like properties [20, 21]. Although some of the ingredients did not satisfy the above criteria, they were still included if their good pharmacological properties were confirmed through a literature review. Finally, the canonical SMILES of the collected active ingredients were entered into the SwissTargetPrediction database (https://www.swisstargetprediction.ch/) to obtain the corresponding target genes. The criterion for target screening was top 100 with a probability greater than 0.1.

#### 2.3.2. Colitis Target Prediction and Intersection With the Targets of the FLCWK Capsule

Information regarding colitis-associated target genes was obtained from the DisGeNET (https://www.disgenet.org/) and GeneCards (https://www.genecards.org/) databases by entering the keyword “Colitis”. The targets of the FLCWK active ingredients and the colitis-related targets were intersected by the Venny 2.1.0 software (https://bioinfogp.cnb.csic.es/tools/venny/) to obtain the potential targets for FLCWK against colitis.

#### 2.3.3. Protein-Protein Interaction (PPI) Network Construction

In order to clarify the interaction of therapeutic target genes and identify the central gene, the potential targets for FLCWK against colitis were imported into the STRING platform (https://string-db.org/) to obtain the PPI network. The species was set as homosapiens, and the parameter was set to the highest confidence (0.900). Then, the Cytoscape 3.10.1 software (https://cytoscape.org/) was used to visualize the PPI network structure and to analyze the topological characteristics.

#### 2.3.4. Gene Ontology (GO) and Kyoto Encyclopedia of Genes and Genomes (KEGG) Enrichment Analysis

The potential targets for FLCWK against colitis were imported into the DAVID database (https://david.ncifcrf.gov/). Then, GO analysis was performed to demonstrate the roles of the potential targets in the biological process (BP), cell composition (CC), and molecular function (MF) of colitis. KEGG analysis was conducted to relate the targets to signaling pathways. The species was set to homosapiens.

#### 2.3.5. Network Construction

The potential targets for FLCWK against colitis and pathways enriched by KEGG analysis were taken into the Cytoscape 3.10.1 software to construct a compound-target-pathway-disease network.

## 3. Results and Discussion

### 3.1. Analysis of the FLCWK Capsule Chemical Components

**Figure 1:**
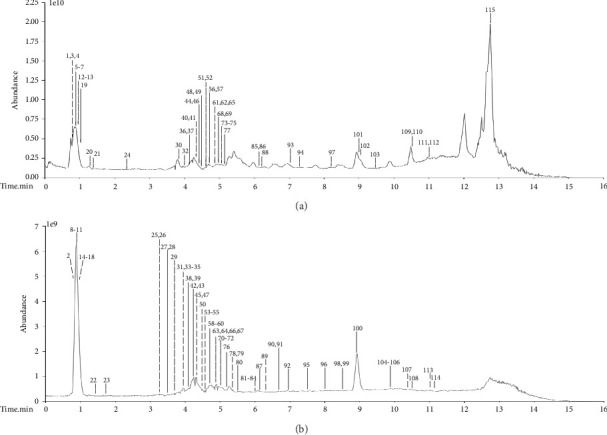
Total ion chromatogram (TIC) of the FLCWK capsule with positive (a) and negative (b) electrospray ionization (ESI).

Based on the UPLC-Q-Exactive Orbitrap MS conditions of “2.2.2”, 115 compounds in the FLCWK capsule were identified under the positive and negative ion modes, including 37 flavonoids, 15 terpenes, 10 fatty acid and derivatives, 9 glycosides, 8 carbohydrates, 7 phenylpropanoids, 5 alkaloids, 5 amino acids, 4 phenols, 4 carboxylic acid and derivatives, four organic acids and derivatives, 3 nucleotides and derivatives, and four others. The total ion chromatograms of the FLCWK capsule are shown in Figure 1. The compound lists are shown in Table 1. The top 7 kinds of compounds with the highest content were flavonoids (54.7%), carbohydrates (22.1%), terpenes (9.3%), alkaloids (3.5%), glycosides (2.8%), phenylpropanoids (1.7%), and phenols (0.4%). Based on the content and anticolitis effects according to reference, flavonoids, terpenes, alkaloids, glycosides, phenylpropanoids, and phenols were chosen to explain their identifications in detail by the following illustrative examples.

#### 3.1.1. Identification of Flavonoids

**Figure 2:**
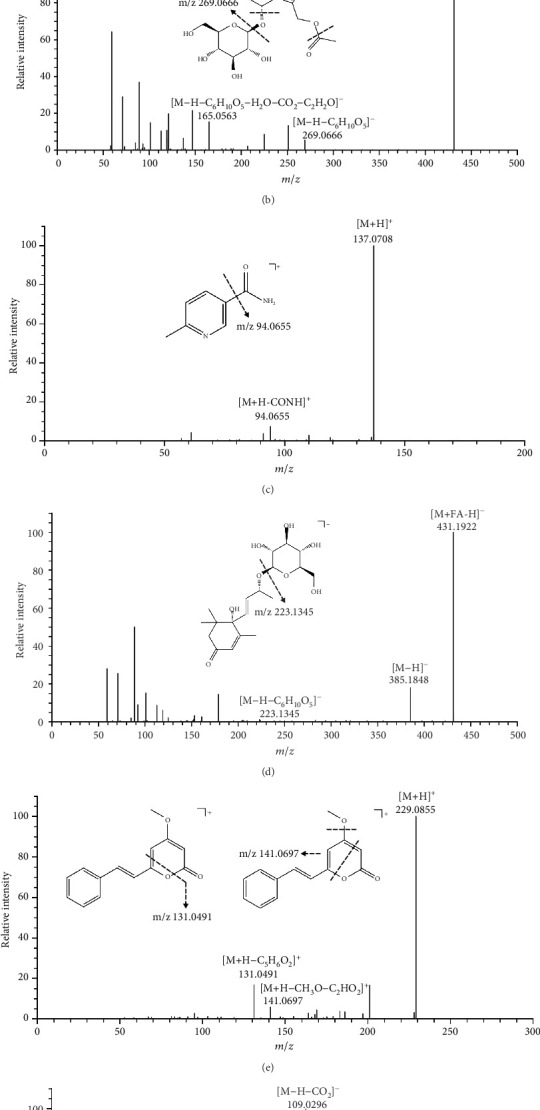
MS/MS spectra and proposed cleavage pathways of rutin (Compound 56) (a), asperulosidic acid (Compound 31) (b), 6-methylnicotinamide (Compound 19) (c), roseoside (Compound 42) (d), desmethoxyyangonin (Compound 101) (e), and protocatechuic acid (Compound 25) (f).

Thirty seven flavonoids were identified in the FLCWK capsule, including kaempferol 3-sophoroside-7-glucosid (33), catechin (37), afzelechi (41), epicatechin (43), manghaslin (44), butin-7-O-β-D-glucopyranoside (50), mauritianin (51), quercetin 3-O-neohesperidoside (54), myricetin 3-O-rutinoside (55), rutin (56), and so on. For example, compound 56 had the precursor ion [M + H]^+^ at m/z 611.1596, indicating the formula of C_27_H_30_O_16_. Its MS/MS spectrum shows the ions resulting from the loss of rhamnose at m/z 465.1023 [M + H-C_6_H_10_O_4_]^+^ and of glucose-rhamnose at m/z 303.0495 [M + H-C_6_H_10_O_4_-C_6_H_10_O_5_]^+^ (Figure 2(a)). By comparison with reference compounds, compound 56 was assigned as rutin.

#### 3.1.2. Identification of Terpenes

Fifteen terpenes were identified in the FLCWK capsule, including geniposidic acid (26), mussaenosidic acid (27), 8-epi-loganic acid-6′-O-beta-D-glucoside (28), asperulosidic acid (31), and so on. Compound 31 showed the deprotonated molecule [M − H]^−^ at m/z 431.1192, indicating the formula of C_18_H_24_O_12_. The fragment ion m/z 269.0666 ([M − H-C_6_H_10_O_5_]^−^) was formed by [M-H]^−^ removing a molecule of glucose. The fragment ion m/z 165.0563 ([M − H-C_6_H_10_O_5_-H_2_O-CO_2_-C_2_H_2_O]^−^) was generated by eliminating H_2_O, CO_2_, and C_2_H_2_O from m/z 269.0666 in succession (Figure 2(b)). The proposed fragmentation pathway of compound 31 was in accordance with the reference compound of asperulosidic acid.

#### 3.1.3. Identification of Alkaloids

Five alkaloids were identified in the FLCWK capsule, including stachydrine (12), 6-methylnicotinamide (19), 4,12-dimethyl-14,19-dioxa-17-azaheptacyclo[10.7.2.22, 5.02, 7.08, 18.08, 21.013, 17]tricosane-4,20-diol (57), songoramine (74), and pellitorine (110). Compound 19 showed the precursor ion [M + H]^+^ at m/z 137.0708, indicating the formula of C_7_H_8_N_2_O. The fragment ion m/z 94.0655 ([M + H-CONH]^+^) was generated by eliminating CONH from the precursor ion (Figure 2(c)). By comparison with the fragmentation pathway of the reference compound, compound 19 was assigned as 6-methylnicotinamide.

#### 3.1.4. Identification of Glycosides

Nine glycosides were identified in the FLCWK capsule, including 4-O-beta-glucopyranosyl-cis-coumaric acid (29), trans-ferulic acid-4-beta-glucoside (34), syringin (36), 2-[4,5-dihydroxy-2-(hydroxymethyl)-6-[(5-methyl-2-propan-2-yl-2H-furan-5-yl)oxy]oxan-3-yl]oxy-6-(hydroxymethyl)oxane-3,4,5-triol (39), roseoside (42), and so on. Compound 42 showed the precursor ion [M + FA-H]^−^ at m/z 431.1922 and [M − H]^−^ at m/z 385.1848, indicating the molecular formula of C_19_H_30_O_8_. The fragment ion m/z 223.1345 ([M − H-C_6_H_10_O_5_]^−^) was formed by [M-H]^−^ removing a molecule of glucose (Figure 2(d)). By comparison with the fragmentation pathway of the reference compound, compound 42 was assigned as roseoside.

#### 3.1.5. Identification of Phenylpropanoids

Seven phenylpropanoids were identified in the FLCWK capsule, including oxyresveratrol 2-O-beta-D-glucopyranoside (40), lyoniresinol 9′-O-glucoside (53), L-3-phenyllactic acid (71), syringaresinol (88), 2-methoxycinnamaldehyde (94), p-hydroxyphenethyl trans-ferulate (97), and desmethoxyyangonin (101). Compound 101 showed the precursor ion [M + H]^+^ at m/z 229.0855, indicating the molecular formula of C_14_H_12_O_3_. As shown in Figure 2(e), the fragment ion m/z 141.0697 ([M + H-CH_3_O-C_2_HO_2_]^+^) was generated by eliminating CH_3_O and C_2_HO_2_ from the precursor ion. The fragment ion m/z 131.0491 ([M + H-C_5_H_6_O_2_]^+^) was formed by [M + H]^+^ removing C_5_H_6_O_2_ (Figure 2(e)). By comparison with the fragmentation pathway of the reference compound, compound 101 was assigned as desmethoxyyangonin.

#### 3.1.6. Identification of Phenols

Four phenols were identified in the FLCWK capsule, including gallic acid (23), protocatechuic acid (25), isovanillic acid (46), and ellagic acid (67). Compound 25 showed a deprotonated molecule [M − H]^−^ peak at m/z 153.0195, indicating the molecular formula of C_7_H_6_O_4_. The deprotonated molecule lost a CO_2_ moiety to form a fragment ion [M − H-CO_2_]^−^ at m/z 109.0296. Then, it was dehydrated to form the [M − H-CO_2_-H_2_O]^−^ fragment ion of m/z 91.0301. The fragmentation pathways of compound 25 are shown in Figure 2(f). Compound 25 was identified as protocatechuic acid by comparing its MS/MS fragmentation pattern and retention time of the reference standard.

### 3.2. Network Pharmacology

#### 3.2.1. Screening of Active Ingredients and Potential Targets of the FLCWK Capsule

The active constituents of the FLCWK capsule, characterized by favorable drug-like and pharmacokinetic properties, were identified utilizing the SwissADME platform. Compounds that did not fully meet the predefined criteria were also included if their pharmacological activity was substantiated by literature evidence. After removing compounds without targets and duplicate potential targets, 46 active ingredients were successfully screened, and their 551 potential targets were predicted by the SwissTargetPrediction platform. More than half of these ingredients are flavonoids, which aligns with the previous literature indicating that the active ingredients of FLCWK contain no less than 12% flavonoids by weight [22]. Experimental pharmacological studies have confirmed that these flavonoids, such as apigenin, quercetin, and quercitrin, exhibit anticolitis effects by suppressing inflammatory mediators [23]. Morin has been shown to alleviate DSS-induced ulcerative colitis in mice through the inhibition of inflammation and modulation of intestinal microbiota [24]. Alpinetin is associated with a dose-dependent reduction in intestinal inflammation and oxidative stress, and it also regulates the expression of tight junctions between cells in ulcerative colitis mice [25]. In addition to flavonoids, the remaining active ingredients include terpenes, phenylpropanoids, phenols, and alkaloids. These compounds are also crucial components of FLCWK, and experimental pharmacological studies have demonstrated that they exhibit a wide range of anti-inflammatory biological activities [26–28]. The information on the active ingredients of the FLCWK capsule is shown in Table 2.

#### 3.2.2. Colitis Target Prediction and Intersection With Targets of the FLCWK Capsule

**Figure 3:**
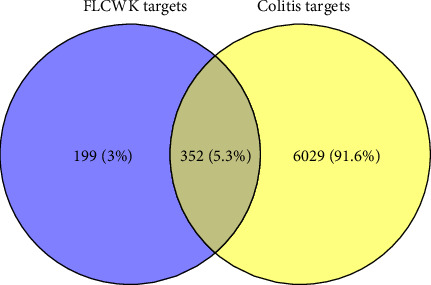
Venn diagram of targets of the FLCWK capsule and colitis.

1135 and 6086 colitis-related targets were collected from the DisGeNET and GeneCards databases, respectively. After combining the results and removing duplicates, 6381 target genes were obtained. 352 potential targets for FLCWK against colitis were obtained after intersecting the targets of the FLCWK active ingredients and the colitis-related targets by the Venny software (Figure 3).

#### 3.2.3. PPI Network Construction

**Figure 4:**
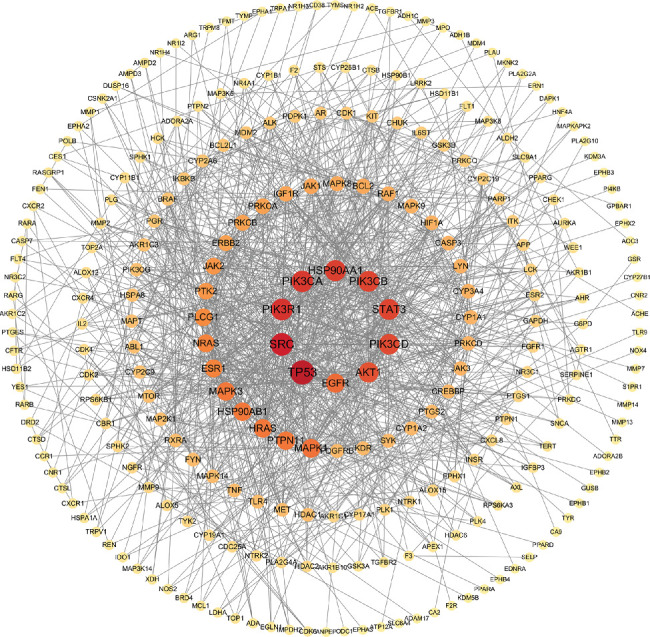
PPI network of FLCWK against colitis. The node became larger, and its color changed from yellow to red with the increased degree of the targets. Network nodes represent proteins. Edges represent protein-protein associations.

Three hundred and fifty-two potential targets for FLCWK against colitis were imported into the STRING database to get PPI information. After removing disconnected targets, a PPI network with 259 nodes and 1066 edges was constructed by the Cytoscape software (Figure 4). In the PPI network, each target was represented by a node, and the interactions between the targets were represented by the edges linking the nodes. A node degree value indicates the number of connections of each node, and the larger a node's degree is, the more it interacts with others. The top 10 targets according to their degree value were TP53 (degree = 49), SRC (degree = 44), PIK3R1 (degree = 41), PIK3CA (degree = 40), HSP90AA1 (degree = 39), STAT3 (degree = 38), PIK3CB (degree = 38), PIK3CD (degree = 37), AKT1 (degree = 36), and EGFR (degree = 32). These may be the key targets when the FLCWK capsule treats colitis.

#### 3.2.4. GO and KEGG Enrichment Analysis

**Figure 5:**
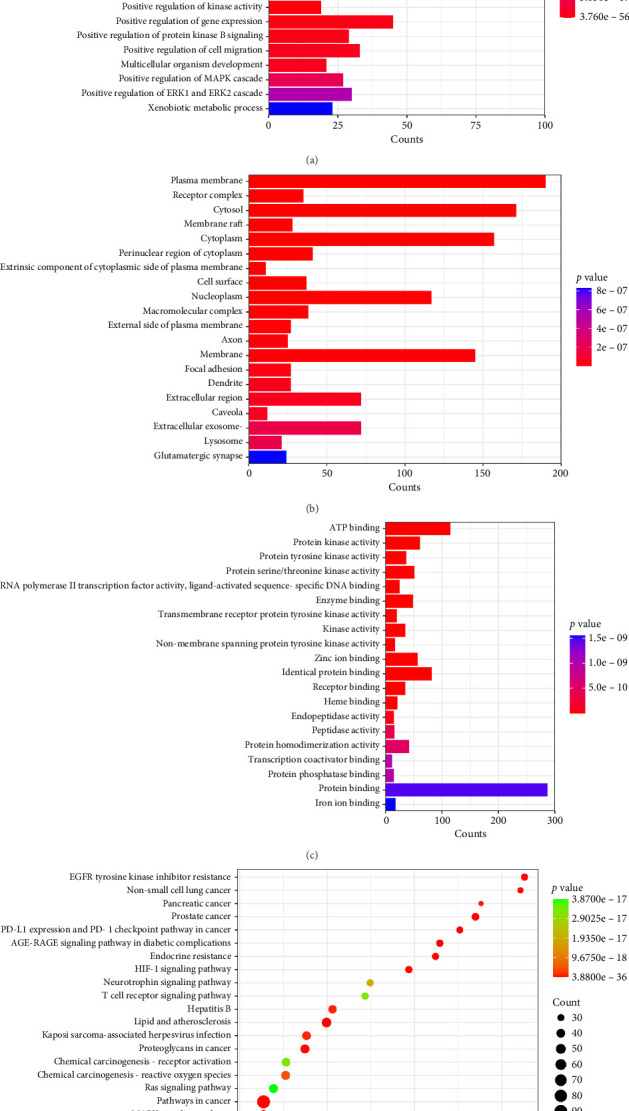
GO and KEGG pathway enrichment analyses of the potential targets for FLCWK against colitis. The bar chart of top 20 BP (a), CC (b), and MF (c) and the bubble chart of top 20 KEGG signaling pathways (d). The redder the color, the more significant the value.

GO and KEGG pathways (*p* < 0.05) were considered significantly enriched. A total of 1042 significantly enriched GO entries were obtained from the DAVID database, including 763 BP, 86 CC, and 193 MF. For visual analysis, the results of GO and KEGG were drawn into bar and bubble charts using a bioinformatics analysis platform (https://www.bioinformatics.com.cn/). As shown in Figure 5(a), 5(b), 5(c), 5(d) and Tables S2-S3, the top 20 GO entries and KEGG pathways were chosen according to the *p* value and counts of hit genes. BP mainly involves protein phosphorylation, response to xenobiotic stimulus, regulation of the apoptotic process, inflammatory response, and so on. CC mainly involves the plasma membrane, receptor complex, cytosol, membrane raft, cytoplasm, and so on. MF mainly involves ATP binding, protein kinase activity, RNA polymerase II transcription factor activity, ligand-activated sequence-specific DNA binding, and so on. Pathogenesis of colitis is always regulated through protein phosphorylation [29]. Furthermore, when the intestinal mucosa is exposed to xenobiotic stimuli, it may produce aberrant responses, resulting in significant inflammation and intestinal damage, such as colitis [30]. Excessive apoptosis of intestinal epithelial cells can lead to epithelial dysfunction and gut microbiology imbalance, which also play an important role in the pathogenesis and progression of colitis [31]. The results of GO suggested that FLCWK may play a role in anticolitis treatment by regulating the abovementioned biological processes via effecting ATP binding, protein kinase, RNA polymerase, and DNA binding. In the KEGG analysis, a total of 164 pathways were enriched, including pathways in cancer, EGFR tyrosine kinase inhibitor resistance, AGE-RAGE, PI3K-Akt, MAPK signaling pathway, and so on. The top 20 pathways were taken to construct the compound-target-pathway-disease network for further analysis of the anticolitis mechanism of the FLCWK capsule.

#### 3.2.5. Network Construction

**Figure 6:**
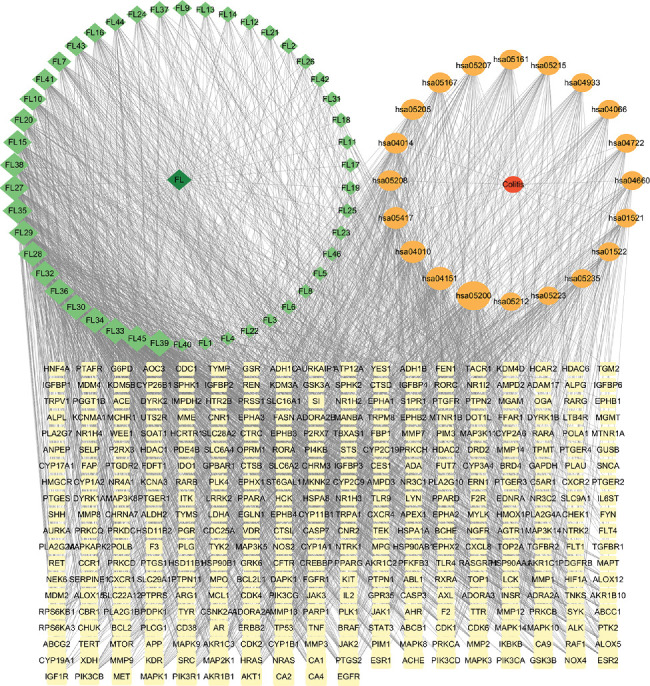
Compound-target-pathway-disease network. Diamonds, ellipses, and rectangles represent active ingredients in the FLCWK capsule, pathways, and target genes, respectively. Each ingredient was linked with its potential pathways and targets. The node became larger with the increased degree of the ingredients, pathways, and target genes.

The compound-target-pathway-disease network was constructed and analyzed by Cytoscape. As shown in Figure 6, active ingredients and their corresponding targets were represented by nodes, and each ingredient was linked to its target genes with edges. Using the Network Analyzer in the Cytoscape software, the topological parameters of the network were calculated. Among the topological parameters, the degree, which refers to the number of edges associated with a node, was selected as a measure of node importance. According to the results of the topological analysis, 4′,5-dihydroxyflavone, pinostrobin, naringenin chalcone, apigenin, morin, and alpinetin were among the top 10 important compounds, suggesting significant anticolitis effects (Table 3). The top 10 core protein targets (Table 4), including EGFR, AKT1, PIK3R1, PIK3CB, MAPK1, IGF1R, and MET, were partially consistent with the results of PPI analysis. Pathways in cancer, MAPK, and PI3K-Akt signaling pathways were among the 10 important pathways in this network (Table 5). These compounds may primarily bind to these core targets to regulate relevant pathways, thereby inhibiting the development of colitis.

Colitis is characterized by chronic relapsing inflammation with intestinal epithelial injury and immune homeostasis disruption [32]. The MAPK signaling pathway is a classical inflammatory signaling pathway, while the PI3K-Akt signaling pathway can activate NF-κB and increase proinflammatory cytokine production (e.g., IL-6, IL-1β, and TNF-α), playing a critical role in colitis pathogenesis [33–35]. Network pharmacology results showed that the compounds of the FLCWK capsule interact with key targets in the MAPK and PI3K-Akt signaling pathways. For example, pinostrobin binds to critical MAPK pathway genes (SRC, FGFR1, and MAPKAPK2) and interacts with key targets in the PI3K-AKT signaling cascade (PIK3CA, PIK3CB, PIK3CD, PIK3CG, MTOR, and AKT1). The previous literature reported that pinostrobin attenuates azoxymethane-induced bowel inflammation in rats [36]. Thus, pinostrobin may alleviate bowel inflammation by inhibiting the MAPK and PI3K-AKT signaling pathways. Apigenin interacts with key MAPK signaling cascade targets (EGFR, SRC, and PIK3R1) and binds to critical PI3K-AKT pathway genes (PIK3R1, AKT1, and GSK3B). These findings are consistent with the previous literature demonstrating that apigenin downregulates inflammatory cytokine expression by modulating the MAPK pathway and inhibits the PI3K-AKT pathway, supporting its anti-inflammatory potential [37]. Morin interacts with crucial MAPK pathway genes (SRC and MAPK) and pivotal PI3K-AKT signaling cascade targets (PIK3R1, PIK3CG, and GSK3B). This is consistent with previous research showing that morin intervention mitigates ulcerative colitis severity in mice by suppressing the MAPK pathways [24]. Alpinetin interacts with a crucial MAPK pathway gene (MAPKAPK2) and key PI3K-AKT pathway targets (PIK3CD, PIK3CB, PIK3CG, PIK3CA, and MTOR). Previous studies indicated that alpinetin improves the disease activity index, colonic shortening, histological scores, and myeloperoxidase activity in mice with ulcerative colitis [25]. Therefore, alpinetin may inhibit colitis via the PI3K-AKT and MAPK signaling pathways. In conclusion, interaction with key targets in the MAPK and PI3K-Akt signaling pathways may be one of the mechanisms by which the FLCWK capsule attenuates the inflammatory response in colitis.

## 4. Conclusions

In this study, an integrated approach combining UHPLC-Q-Exactive Orbitrap MS and network pharmacology analysis was adopted to explore the potential active ingredients and anticolitis mechanisms of the FLCWK capsule. 115 compounds in the FLCWK capsule were identified. According to the results of the compound-target-pathway-disease network, the anticolitis effect of the FLCWK capsule is mainly attributed to 46 active ingredients such as 4′,5-dihydroxyflavone, pinostrobin, naringenin chalcone, apigenin, and morin, which act on 352 core protein targets, such as EGFR, AKT1, PIK3R1, PIK3CB, and MAPK1, thereby modulating relevant pathways, such as MAPK and PI3K-Akt signaling pathways. In conclusion, the integrated approach provided valuable insights into the potential active ingredients and anticolitis mechanisms of the FLCWK capsule. Based on the current findings, further confirmation through in vitro and in vivo experiments in subsequent studies is required to establish a reliable foundation for its clinical application.

## Figures and Tables

**Table 1:** Chemical components characterized from the FLCWK capsule.

No.	Retention time (min)	Compound	Molecular formula	Selective ion	Theoretical mass (m/z)	Measured mass (m/z)	Error (ppm)	Major fragments (m/z)	Classification
1	0.77	Mannosamine	C_6_H_13_NO_5_	[M + H − H_2_O]^+^	162.0761	162.0759	−1.05	72.0451, 84.0449, 84.9602, 85.0289, 97.0288, 102.9706, 120.9810, 143.9967, 144.0654	Carbohydrates
2	0.77	Iminodiacetic acid	C_4_H_7_NO_4_	[M − H]^−^	132.0302	132.0303	0.68	86.0248, 88.0405	Amino acids
3	0.78	L-Histidine	C_6_H_9_N_3_O_2_	[M + H]^+^	156.0768	156.0766	−1.23	56.9655, 74.0971, 95.0608, 110.0714, 130.0630	Amino acids
4	0.78	L-Arginine	C_6_H_14_N_4_O_2_	[M + H]^+^	175.1190	175.1188	−1.04	72.0814, 112.0857, 116.0708, 130.0500, 130.0974, 132.0211, 139.0389, 158.0921	Amino acids
5	0.82	6-(alpha-D-Glucosaminyl)-1D-myo-inositol	C_12_H_23_NO_10_	[M + H]^+^	342.1395	342.1390	−1.40	144.0656, 162.076, 174.076, 240.0862, 288.1073, 306.1182	Carbohydrates
6	0.85	3-Pyridineacetic acid	C_7_H_7_NO_2_	[M + H]^+^	138.0550	138.0547	−1.61	56.9656, 110.0603	Carboxylic acid and derivatives
7	0.87	Melezitose	C_18_H_32_O_16_	[M + Na]^+^	527.1583	527.1576	−1.39	185.0421, 203.0524, 347.0944, 365.1052	Carbohydrates
8	0.88	Turanose	C_12_H_22_O_11_	[M + FA − H]^−^	387.1145	387.1141	−0.98	59.0139, 71.014, 89.0245, 101.0245, 113.0244, 119.035, 179.0557, 341.1085	Carbohydrates
9	0.88	Gluconic acid	C_6_H_12_O_7_	[M − H]^−^	195.0510	195.0510	−0.12	59.0139, 75.0087, 85.0293, 87.0088, 89.0245, 99.0087, 129.0191, 159.0298, 177.0406	Carbohydrates
10	0.88	Gentianose	C_18_H_32_O_16_	[M + FA − H]^−^	549.1673	549.1669	−0.65	143.0347, 161.0456, 179.0561, 191.0558, 207.0509, 221.0662, 323.0978, 341.1090, 503.1613	Carbohydrates
11	0.88	Threonic acid	C_4_H_8_O_5_	[M − H]^−^	135.0299	135.0300	0.76	87.0087, 89.0245, 92.0255, 108.0453, 116.0074, 134.0176	Carbohydrates
12	0.89	Stachydrine	C_7_H_13_NO_2_	[M + K]^+^	182.0578	182.0576	−0.98	84.9602, 120.0808, 166.0836	Alkaloids
13	0.89	FAPy-adenine	C_5_H_7_N_5_O	[M + H − H_2_O]^+^	136.0618	136.0617	−0.73	91.0547,109.0649,119.0355	Others
14	0.90	Glucose	C_6_H_12_O_6_	[M − H]^−^	179.0561	179.0562	0.68	72.9932, 75.0087, 85.0294, 87.0087, 89.0245, 95.0140, 101.0251, 113.0247, 119.0350	Carbohydrates
15	0.90	Glycolaldehyde dimer	C_4_H_8_O_4_	[M − H]^−^	119.0350	119.0351	0.74	71.0139, 72.0092, 72.9932, 73.0296, 74.0248, 75.0088, 89.0245, 101.0245, 118.0509	Others
16	0.92	Citric acid	C_6_H_8_O_7_	[M − H]^−^	191.0197	191.0199	0.81	71.0136, 85.0296, 87.0088, 93.0346, 111.0088, 111.0452, 127.0403, 129.0194, 173.0457	Organic acids and derivatives
17	0.93	Malic acid	C_4_H_6_O_5_	[M − H]^−^	133.0142	133.0144	1.17	71.0139, 74.0248, 114.934, 115.0037	Organic acids and derivatives
18	0.93	Uridine	C_9_H_12_N_2_O_6_	[M − H]^−^	243.0623	243.0622	−0.41	99.9259, 110.0239, 153.0305	Nucleotides and derivatives
19	1.04	6-Methylnicotinamide	C_7_H_8_N_2_O	[M + H]^+^	137.0709	137.0708	−1.34	94.0655	Alkaloids
20	1.32	L-Isoleucine	C_6_H_13_NO_2_	[M + H]^+^	132.1019	132.1017	−1.20	69.0705, 72.9378, 86.0969, 90.0554	Amino acids
21	1.39	Adenosine	C_10_H_13_N_5_O_4_	[M + H]^+^	268.1040	268.1035	−2.13	136.0617	Nucleotides and derivatives
22	1.41	Methylmalonic acid	C_4_H_6_O_4_	[M − H]^−^	117.0193	117.0195	1.40	59.0139, 71.0503, 72.0092, 73.0295, 74.0248, 99.0089, 99.9258, 116.9286	Organic acids and derivatives
23	1.82	Gallic acid	C_7_H_6_O_5_	[M − H]^−^	169.0142	169.0144	1.11	98.0247, 125.0245	Phenols
24	2.28	N-(1-Deoxy-1-fructosyl)phenylalanine	C_15_H_21_NO_7_	[M + H]^+^	328.1391	328.1383	−2.50	120.0809, 132.0807, 166.0863, 178.0865, 264.1225, 292.1174, 310.1281	Amino acids
25	3.29	Protocatechuic acid	C_7_H_6_O_4_	[M − H]^−^	153.0193	153.0195	1.00	91.0301, 109.0296	Phenols
26	3.33	Geniposidic acid	C_16_H_22_O_10_	[M − H]^−^	373.1140	373.1137	−0.77	59.0138, 71.0137, 89.0244, 93.0347, 101.0244, 123.0451, 149.0606, 167.0717, 211.0608, 373.1134	Terpenes
27	3.63	Mussaenosidic acid	C_16_H_24_O_10_	[M − H]^−^	375.1297	375.1296	−0.23	89.0243, 101.0241, 102.9566, 121.0662, 125.0609, 149.0603, 151.0768, 169.0877, 213.0766	Terpenes
28	3.69	8-Epi-loganic acid-6′-O-beta-D-glucoside	C_22_H_34_O_15_	[M − H]^−^	537.1825	537.1824	−0.09	250.1856, 307.1030, 310.4752, 327.3016, 382.9057, 454.2435, 459.8475, 491.1820, 491.2547	Terpenes
29	3.80	4-O-beta-glucopyranosyl-cis-coumaric acid	C_15_H_18_O_8_	[M + FA − H]^−^	371.0984	371.0981	−0.90	96.9888, 110.0945, 119.0501, 134.0316, 134.2957, 136.3259, 147.0451, 163.0401, 342.3176	Glycosides
30	3.81	5′-Methylthioadenosine	C_11_H_15_N_5_O_3_S	[M + H]^+^	298.0968	298.0962	−2.20	136.0617	Nucleotides and derivatives
31	3.95	Asperulosidic acid	C_18_H_24_O_12_	[M − H]^−^	431.1195	431.1192	−0.71	165.0563, 269.0666	Terpenes
32	3.99	3,17-dihydroxy-4,4,8,10,14-pentamethyl-2,3,5,6,7,9-hexahydro-1H-cyclopenta[a]phenanthrene-15,16-dione	C_22_H_30_O_4_	[M + NH_4_]^+^	376.2483	376.2477	−1.41	225.1230,358.2383	Terpenes
33	4.05	Kaempferol 3-sophoroside-7-glucoside	C_33_H_40_O_21_	[M − H]^−^	771.1989	771.1990	0.05	299.0192, 300.0272, 301.0349, 462.0822, 609.1448	Flavonoids
34	4.05	*Trans*-ferulic acid-4-beta-glucoside	C_16_H_20_O_9_	[M − H_2_O − H]^−^	337.0929	337.0927	−0.56	119.0502, 163.0401, 173.0453, 191.0562	Glycosides
35	4.10	Crenulatin	C_11_H_20_O_6_	[M + FA − H]^−^	293.1242	293.1241	−0.21	59.0139, 71.0138, 89.0246, 101.0245, 113.0243, 119.0347, 131.0714, 157.0119	Terpenes
36	4.12	Syringin	C_17_H_24_O_9_	[M + Na]^+^	395.1313	395.1307	−1.45	185.0424,232.0702,233.0772	Glycosides
37	4.17	Catechin	C_15_H_14_O_6_	[M + H]^+^	291.0863	291.0858	−1.93	111.0806, 113.0962, 123.0442, 127.0389, 129.0911, 139.0388, 145.0496, 147.0437, 165.0545	Flavonoids
38	4.23	Asperuloside	C_18_H_22_O_11_	[M + FA − H]^−^	459.1144	459.1144	−0.03	59.0139, 119.0502, 147.0452, 191.035, 413.1062	Terpenes
39	4.23	2-[4,5-dihydroxy-2-(hydroxymethyl)-6-[(5-methyl-2-propan-2-yl-2H-furan-5-yl)oxy]oxan-3-yl]oxy-6-(hydroxymethyl)oxane-3,4,5-triol	C_20_H_34_O_12_	[M + FA − H]^−^	511.2033	511.2029	−0.72	71.0139, 89.0245, 92.5903, 101.0243, 388.8512, 465.1975	Glycosides
40	4.31	Oxyresveratrol 2-O-beta-D-glucopyranoside	C_20_H_22_O_9_	[M + H]^+^	407.1337	407.1330	−1.51	149.0597, 163.0389, 205.0492, 235.0597, 253.0703, 257.0805, 271.0811, 275.0911, 406.2216	Phenylpropanoids
41	4.32	Afzelechin	C_15_H_14_O_5_	[M + H]^+^	275.0914	275.0908	−2.03	107.0494, 111.0807, 127.0756, 129.0908, 139.0389, 149.0598, 191.0704	Flavonoids
42	4.38	Roseoside	C_19_H_30_O_8_	[M + FA − H]^−^	431.1923	431.1922	−0.26	223.1345, 385.1848	Glycosides
43	4.40	Epicatechin	C_15_H_14_O_6_	[M − H]^−^	289.0718	289.0718	0.02	137.0245, 151.0412, 161.0609, 179.0364, 187.0404, 203.0710, 205.0509, 221.0823, 245.0814	Flavonoids
44	4.45	Manghaslin	C_33_H_40_O_20_	[M + H]^+^	757.2186	757.2172	−1.78	71.0499, 85.029, 129.0548, 287.0546, 303.0496, 449.1082, 465.1024, 611.1595	Flavonoids
45	4.45	2-[2-Hydroxy-4-(3-hydroxybut-1-enyl)-3,5,5-trimethylcyclohex-3-en-1-yl]oxy-6-(hydroxymethyl)oxane-3,4,5-triol	C_19_H_32_O_8_	[M + FA − H]^−^	433.2079	433.2080	0.14	89.0246, 92.5869, 101.0245, 113.0246, 119.0350, 179.0562, 225.1486, 366.1724, 387.2025	Glycosides
46	4.45	Isovanillic acid	C_8_H_8_O_4_	[M + H]^+^	169.0495	169.0493	−1.33	141.0546, 146.9613, 146.9804, 151.0390, 151.0755, 151.0864, 168.0641	Phenols
47	4.45	1,3,4-Trihydroxy-5-[3-(4-hydroxyphenyl)prop-2-enoyloxy]cyclohexane-1-carboxylic acid	C_16_H_18_O_8_	[M − H]^−^	337.0929	337.0930	0.18	93.0347, 111.0452, 113.0245, 119.05, 162.0555, 163.0403, 173.0455, 191.0563	Organic acids and derivatives
48	4.52	Megastigm-7-ene-3,5,6,9-tetraol	C_13_H_24_O_4_	[M + H − H_2_O]^+^	227.1641	227.1638	−1.66	177.0549, 181.0489, 181.9503, 186.9560, 191.1428, 199.0868, 209.0795	Terpenes
49	4.54	Paeonolide	C_20_H_28_O_12_	[M + Na]^+^	483.1473	483.1465	−1.77	331.0988	Glycosides
50	4.57	Butin-7-O-β-D-glucopyranoside	C_21_H_22_O_10_	[M − H_2_O − H]^−^	415.1035	415.1037	0.47	137.0249, 149.0244, 149.0605, 203.0721, 205.0511, 215.0726, 245.0822, 289.0715, 301.0698	Flavonoids
51	4.59	Mauritianin	C_33_H_40_O_19_	[M + H]^+^	741.2237	741.2222	−2.01	71.0498, 85.029, 129.0547, 287.0547, 449.1079, 595.1638	Flavonoids
52	4.59	Neridienone A	C_21_H_26_O_3_	[M + NH_4_]^+^	344.2220	344.2220	−0.14	308.2006, 326.211	Terpenes
53	4.66	Lyoniresinol 9′-O-glucoside	C_28_H_38_O_13_	[M + FA − H]^−^	627.2294	627.2295	0.05	205.0710, 359.1140, 371.1140, 373.1273, 389.1249, 404.1474, 419.1715, 534.2642, 535.2736, 581.2214	Phenylpropanoids
54	4.70	Quercetin 3-O-neohesperidoside	C_27_H_30_O_16_	[M − H]^−^	609.1461	609.1460	−0.17	151.0038, 255.0301, 271.0244, 300.0272	Flavonoids
55	4.70	Myricetin 3-O-rutinoside	C_27_H_30_O_17_	[M − H]^−^	625.1410	625.1408	−0.36	92.5929, 151.0039, 161.0237, 178.9989, 255.0290, 271.0243, 287.0197, 299.0197, 316.0216	Flavonoids
56	4.70	Rutin	C_27_H_30_O_16_	[M + H]^+^	611.1607	611.1596	−1.81	303.0495, 465.1023	Flavonoids
57	4.72	4,12-dimethyl-14,19-dioxa-17-azaheptacyclo[10.7.2.22,5.02,7.08,18.08,21.013,17]tricosane-4,20-diol	C_22_H_33_NO_4_	[M + H − H_2_O]^+^	358.2376	358.2370	−1.74	357.2251	Alkaloids
58	4.77	Butyl (S)-3-hydroxybutyrate [arabinosyl-(1-> 6)-glucoside]	C_19_H_34_O_12_	[M − H]^−^	453.1978	453.1977	−0.02	103.0400, 112.9857, 113.0246, 119.0346, 143.0356, 163.0608, 205.0713, 248.9588, 407.1904	Glycosides
59	4.81	Quercetin-3-O-glucuronide	C_21_H_18_O_13_	[M − H]^−^	477.0675	477.0673	−0.34	71.0138, 89.0245, 92.5887, 119.0503, 149.0608, 151.0039, 163.0401, 178.9987, 301.0352	Flavonoids
60	4.81	Nuciferoside	C_22_H_38_O_11_	[M + FA − H]^−^	523.2396	523.2394	−0.40	152.9955, 161.0455, 163.0608, 165.0557, 179.0722, 205.0730, 331.1747, 361.1655, 477.2361	Glycosides
61	4.83	Hyperoside	C_21_H_20_O_12_	[M + H]^+^	465.1028	465.1021	−1.46	85.029, 303.0497	Flavonoids
62	4.83	Myricitrin	C_21_H_20_O_12_	[M + Na]^+^	487.0847	487.0838	−1.87	325.0312	Flavonoids
63	4.85	Apigenin 5-O-glucoside	C_21_H_20_O_10_	[M − H]^−^	431.0984	431.0988	0.91	113.0236, 119.0348, 181.0330, 205.1233, 269.0452, 283.0605, 311.0549, 341.0659, 430.1841	Flavonoids
64	4.85	Quercetin 3-o-(6″-galloyl)-beta-d-glucopyranoside	C_28_H_24_O_16_	[M − H]^−^	615.0992	615.0992	0.10	151.0037, 178.999, 300.0285, 301.035	Flavonoids
65	4.85	(16-Hydroxy-5,5,9-trimethyl-14-methylidene-15-oxo-2-tetracyclo[11.2.1.01,10.04,9]hexadecanyl) acetate	C_22_H_32_O_4_	[M + NH_4_]^+^	378.2639	378.2633	−1.70	360.2528, 377.2516	Terpenes
66	4.85	Suberic acid	C_8_H_14_O_4_	[M − H]^−^	173.0819	173.0821	0.79	93.0346, 104.9539, 111.0815, 129.0921, 130.0875, 146.0362, 172.0976	Fatty acid and derivatives
67	4.85	Ellagic acid	C_14_H_6_O_8_	[M − H]^−^	300.9990	300.9990	0.06	92.5915	Phenols
68	4.88	*m*-Anisaldehyde	C_8_H_8_O_2_	[M + H]^+^	137.0597	137.0595	−1.37	95.0861, 96.0451, 109.0650, 110.0604, 120.0447, 136.0218, 136.0617, 136.0748	Carboxylic acid and derivatives
69	4.90	Kaempferol-3-O-rutinoside	C_27_H_30_O_15_	[M + H]^+^	595.1657	595.1646	−1.90	71.0498, 85.0289, 287.0546, 449.1073	Flavonoids
70	4.90	Kaempferol 3-O-robinobioside	C_27_H_30_O_15_	[M − H]^−^	593.1512	593.1511	−0.10	227.0353, 255.0265, 284.0323	Flavonoids
71	4.96	L-3-Phenyllactic acid	C_9_H_10_O_3_	[M − H]^−^	165.0557	165.0558	0.68	97.0407, 98.0248, 119.0504, 120.0456, 121.0295, 121.0661, 122.0614, 147.0453	Phenylpropanoids
72	5.03	Kaempferol-3-O-glucuronoside	C_21_H_18_O_12_	[M − H]^−^	461.0725	461.0726	0.04	71.0138, 85.0296, 113.0246, 229.0504, 257.0465, 285.0409	Flavonoids
73	5.05	Trifolin	C_21_H_20_O_11_	[M + Na]^+^	471.0898	471.0890	−1.76	309.0364	Flavonoids
74	5.05	Songoramine	C_22_H_29_NO_3_	[M + H]^+^	356.2220	356.2213	−2.03	338.2112	Alkaloids
75	5.06	Quercitrin	C_21_H_20_O_11_	[M + H]^+^	449.1078	449.1070	−1.85	85.0289, 287.0547, 303.049	Flavonoids
76	5.13	Vitexin	C_21_H_20_O_10_	[M − H]^−^	431.0984	431.0986	0.58	59.0139, 89.0244, 268.0375, 269.0444	Flavonoids
77	5.13	Sophoricoside	C_21_H_20_O_10_	[M + H]^+^	433.1129	433.1123	−1.49	271.0599	Flavonoids
78	5.28	2-(4-methoxyphenyl)-7-[3,4,5-trihydroxy-6-(hydroxymethyl)oxan-2-yl]oxychromen-4-one	C_22_H_22_O_9_	[M + FA − H]^−^	475.1246	475.1245	−0.26	134.0374, 149.0609, 163.0401, 236.0484, 253.0499, 295.0613, 312.0633, 313.1073	Flavonoids
79	5.30	Aloeresin D	C_29_H_32_O_11_	[M − H_2_O − H]^−^	537.1766	537.1749	−3.07	190.0270, 205.0494, 225.1749, 298.7839, 341.1386, 342.3734, 355.4550, 367.1013, 474.4010	Others
80	5.52	Okanin	C_15_H_12_O_6_	[M − H]^−^	287.0561	287.0562	0.24	92.9275, 96.9604, 125.0245, 149.061, 243.0663, 259.0609	Flavonoids
81	5.99	Steppogenin	C_15_H_12_O_6_	[M − H]^−^	287.0561	287.0560	−0.32	107.014, 135.0452, 139.0403, 151.0036	Flavonoids
82	6.01	Chrysophanein	C_21_H_20_O_9_	[M + FA − H]^−^	461.1089	461.1089	−0.13	253.0503	Others
83	6.03	Sebacic acid	C_10_H_18_O_4_	[M − H]^−^	201.1132	201.1133	0.51	74.0249, 89.0243, 116.9285, 139.1129, 183.1023	Fatty acid and derivatives
84	6.07	Luteolin	C_15_H_10_O_6_	[M − H]^−^	285.0405	285.0405	0.05	133.0295	Flavonoids
85	6.08	(5S,10S,13R,14R,15S,17R)-15-hydroxy-17-[(Z,2R)-7-hydroxy-6-methylhept-5-en-2-yl]-4,4,10,13,14-pentamethyl-1,2,5,6,12,15,16,17-octahydrocyclopenta[a]phenanthren-3-one	C_30_H_46_O_3_	[M + NH_4_]^+^	472.3786	472.3776	−2.03	119.0859,454.3658	Terpenes
86	6.10	Quercetin	C_15_H_10_O_7_	[M + H]^+^	303.0499	303.0493	−2.04	229.0494	Flavonoids
87	6.10	Morin	C_15_H_10_O_7_	[M − H]^−^	301.0354	301.0352	−0.49	107.0139, 121.0296, 151.0037, 178.9988	Flavonoids
88	6.25	Syringaresinol	C_22_H_26_O_8_	[M + H − H_2_O]^+^	401.1594	401.1587	−1.90	291.0995, 315.0858, 330.1095, 331.1151, 339.1240, 343.1169, 351.1234, 369.1332, 383.1486	Phenylpropanoids
89	6.37	3-O-Methylquercetin	C_16_H_12_O_7_	[M − H]^−^	315.0510	315.0510	−0.01	255.029, 271.025, 300.0269	Flavonoids
90	6.74	Naringenin chalcone	C_15_H_12_O_5_	[M − H]^−^	271.0612	271.0611	−0.38	93.0347, 107.0139, 119.0504, 151.0037, 227.072	Flavonoids
91	6.79	Apigenin	C_15_H_10_O_5_	[M − H]^−^	269.0455	269.0455	−0.11	117.0347, 149.0242, 151.0035	Flavonoids
92	6.92	Kaempferol	C_15_H_10_O_6_	[M − H]^−^	285.0405	285.0404	−0.32	165.991,270.0542	Flavonoids
93	7.09	Alpinetin	C_16_H_14_O_4_	[M + H]^+^	271.0965	271.0958	−2.47	131.0496, 135.0028, 167.0337	Flavonoids
94	7.28	2-Methoxycinnamaldehyde	C_10_H_10_O_2_	[M + H]^+^	163.0754	163.0750	−2.27	117.0701, 133.0284, 135.0441, 145.0647	Phenylpropanoids
95	7.62	Dodecanedioic acid	C_12_H_22_O_4_	[M − H]^−^	229.1445	229.1444	−0.61	116.9286, 167.1441, 211.1337	Fatty acid and derivatives
96	8.07	3-Hydroxycapric acid	C_10_H_20_O_3_	[M − H]^−^	187.1340	187.1340	0.42	59.014, 61.9882, 125.0972	Carboxylic acid and derivatives
97	8.23	p-Hydroxyphenethyl trans-ferulate	C_18_H_18_O_5_	[M + H]^+^	315.1227	315.1220	−2.33	131.0488, 196.0363, 211.0597	Phenylpropanoids
98	8.65	4′,5-Dihydroxyflavone	C_15_H_10_O_4_	[M − H]^−^	253.0506	253.0506	−0.31	116.9284, 209.1545	Flavonoids
99	8.65	Dihydroartemisinin	C_15_H_24_O_5_	[M − H_2_O − H]^−^	265.1445	265.1442	−1.16	61.9885,129.9759,221.1548	Terpenes
100	8.93	Acacetin	C_16_H_12_O_5_	[M − H]^−^	283.0612	283.0611	−0.42	268.0372	Flavonoids
101	9.08	Desmethoxyyangonin	C_14_H_12_O_3_	[M + H]^+^	229.0859	229.0855	−1.86	131.0491, 141.0697	Phenylpropanoids
102	9.11	L-Borneol	C_10_H_18_O	[M + NH_4_]^+^	172.1696	172.1692	−2.59	126.0914, 130.0498, 130.9665, 145.0984, 148.9768, 149.9401, 171.1491	Terpenes
103	9.46	15-Hydroxydehydroabietic acid	C_20_H_28_O_3_	[M + H − H_2_O]^+^	299.2005	299.1999	−2.01	145.101, 280.2628	Terpenes
104	9.85	Cardamonin	C_16_H_14_O_4_	[M − H]^−^	269.0819	269.0819	−0.23	89.0244, 165.0193, 226.0626, 254.0583	Flavonoids
105	9.89	12,13-Dihydroxyoctadec-9-enoate	C_18_H_34_O_4_	[M − H]^−^	313.2384	313.2384	−0.20	99.082, 129.0923, 183.1388, 201.1134, 295.2281	Fatty acid and derivatives
106	9.89	3-Hydroxydodecanoic acid	C_12_H_24_O_3_	[M − H]^−^	215.1653	215.1652	−0.28	59.0139	Carboxylic acid and derivatives
107	10.30	Hexadecanedioic acid	C_16_H_30_O_4_	[M − H]^−^	285.2071	285.2070	−0.55	223.2065, 267.1961	Fatty acid and derivatives
108	10.41	Pelargonic acid	C_9_H_18_O_2_	2[M − H]^−^	315.2541	315.2538	−0.85	297.2423, 313.2381	Fatty acid and derivatives
109	10.43	Pinostrobin	C_16_H_14_O_4_	[M + H]^+^	271.0965	271.0958	−2.47	131.0492, 167.0338	Flavonoids
110	10.46	Pellitorine	C_14_H_25_NO	[M + NH_4_]^+^	241.2275	241.2268	−2.81	88.0762, 200.2006	Alkaloids
111	10.96	Ricinoleic acid	C_18_H_34_O_3_	[M + H − H_2_O]^+^	281.2475	281.2468	−2.50	175.1479, 179.1429, 179.1785, 189.1632, 193.1585, 207.1742, 221.2258, 245.2260, 263.2365	Fatty acid and derivatives
112	11.04	Lupenone	C_30_H_48_O	[M + H − H_2_O]^+^	407.3672	407.3663	−2.12	109.1015, 121.1015, 123.1170, 135.1169, 147.1167, 149.1317, 189.1642, 203.1792, 335.1665	Terpenes
113	11.04	Octadecanedioic acid	C_18_H_34_O_4_	[M − H]^−^	313.2384	313.2383	−0.38	251.2377, 295.2271, 312.1719	Fatty acid and derivatives
114	11.19	2-Hydroxytetradecanoic acid	C_14_H_28_O_3_	[M − H]^−^	243.1966	243.1964	−0.66	197.1912	Fatty acid and derivatives
115	12.74	Oleamide	C_18_H_35_NO	2[M + H]^+^	563.5510	563.5502	−1.51	69.0707, 83.0862, 97.1017, 247.242, 265.2523, 282.2792	Fatty acid and derivatives

**Table 2:** The information on the active ingredients of the FLCWK capsule.

No.	Compound	GI absorption	Lipinski	Ghose	Veber	Egan	Muegge	Number of yes
FL1	6-Methylnicotinamide	High	Yes	No	Yes	Yes	No	3
FL2	Gallic acid	High	Yes	No	Yes	Yes	No	3
FL3	Protocatechuic acid	High	Yes	No	Yes	Yes	No	3
FL4	Geniposidic acid	Low	Yes	No	No	No	No	1
FL5	3,17-dihydroxy-4,4,8,10,14-pentamethyl-2,3,5,6,7,9-hexahydro-1H-cyclopenta[a]phenanthrene-15,16-dione	High	Yes	Yes	Yes	Yes	Yes	5
FL6	Kaempferol 3-sophoroside-7-glucoside	Low	No	No	No	No	No	0
FL7	Roseoside	Low	Yes	No	Yes	No	Yes	3
FL8	Manghaslin	Low	No	No	No	No	No	0
FL9	Isovanillic acid	High	Yes	Yes	Yes	Yes	No	4
FL10	Neridienone A	High	Yes	Yes	Yes	Yes	Yes	5
FL11	Rutin	Low	No	No	No	No	No	0
FL12	Quercetin-3-O-glucuronide	Low	No	No	No	No	No	0
FL13	Hyperoside	Low	No	No	No	No	No	0
FL14	Myricitrin	Low	No	Yes	No	No	No	1
FL15	(16-Hydroxy-5,5,9-trimethyl-14-methylidene-15-oxo-2-tetracyclo[11.2.1.01,10.04,9]hexadecanyl) acetate	High	Yes	Yes	Yes	Yes	Yes	5
FL16	Ellagic acid	High	Yes	Yes	No	No	Yes	3
FL17	Kaempferol-3-O-rutinoside	Low	No	No	No	No	No	0
FL18	Kaempferol 3-O-robinobioside	Low	No	No	No	No	No	0
FL19	L-3-Phenyllactic acid	High	Yes	Yes	Yes	Yes	No	4
FL20	Songoramine	High	Yes	Yes	Yes	Yes	Yes	5
FL21	Quercitrin	Low	No	Yes	No	No	No	1
FL22	Vitexin	Low	Yes	Yes	No	No	No	2
FL23	Sophoricoside	Low	Yes	Yes	No	No	No	2
FL24	2-(4-methoxyphenyl)-7-[3,4,5-trihydroxy-6-(hydroxymethyl)oxan-2-yl]oxychromen-4-one	High	Yes	Yes	Yes	No	Yes	4
FL25	Okanin	High	Yes	Yes	Yes	Yes	Yes	5
FL26	Steppogenin	High	Yes	Yes	Yes	Yes	Yes	5
FL27	Luteolin	High	Yes	Yes	Yes	Yes	Yes	5
FL28	(5S,10S,13R,14R,15S,17R)-15-hydroxy-17-[(Z,2R)-7-hydroxy-6-methylhept-5-en-2-yl]-4,4,10,13,14-pentamethyl-1,2,5,6,12,15,16,17-octahydrocyclopenta[a]phenanthren-3-one	High	Yes	No	Yes	No	No	2
FL29	Quercetin	High	Yes	Yes	Yes	Yes	Yes	5
FL30	Morin	High	Yes	Yes	Yes	Yes	Yes	5
FL31	Syringaresinol	High	Yes	Yes	Yes	Yes	Yes	5
FL32	3-O-Methylquercetin	High	Yes	Yes	Yes	Yes	Yes	5
FL33	Naringenin chalcone	High	Yes	Yes	Yes	Yes	Yes	5
FL34	Apigenin	High	Yes	Yes	Yes	Yes	Yes	5
FL35	Kaempferol	High	Yes	Yes	Yes	Yes	Yes	5
FL36	Alpinetin	High	Yes	Yes	Yes	Yes	Yes	5
FL37	2-Methoxycinnamaldehyde	High	Yes	Yes	Yes	Yes	No	4
FL38	p-Hydroxyphenethyl trans-ferulate	High	Yes	Yes	Yes	Yes	Yes	5
FL39	4′,5-Dihydroxyflavone	High	Yes	Yes	Yes	Yes	Yes	5
FL40	Dihydroartemisinin	High	Yes	Yes	Yes	Yes	Yes	5
FL41	Acacetin	High	Yes	Yes	Yes	Yes	Yes	5
FL42	L-Borneol	High	Yes	No	Yes	Yes	No	3
FL43	15-Hydroxydehydroabietic acid	High	Yes	Yes	Yes	Yes	Yes	5
FL44	Cardamonin	High	Yes	Yes	Yes	Yes	Yes	5
FL45	Pinostrobin	High	Yes	Yes	Yes	Yes	Yes	5
FL46	Pellitorine	High	Yes	Yes	Yes	Yes	Yes	5

**Table 3:** Top 10 important compounds of the compound-target-pathway-disease network.

No.	Compound	Degree
FL39	4′,5-Dihydroxyflavone	79
FL45	Pinostrobin	76
FL33	Naringenin chalcone	75
FL34	Apigenin	75
FL30	Morin	74
FL36	Alpinetin	74
FL28	(5S,10S,13R,14R,15S,17R)-15-hydroxy-17-[(Z,2R)-7-hydroxy-6-methylhept-5-en-2-yl]-4,4,10,13,14-pentamethyl-1,2,5,6,12,15,16,17-octahydrocyclopenta[a]phenanthren-3-one	72
FL29	Quercetin	72
FL32	3-O-Methylquercetin	72
FL35	Kaempferol	72

**Table 4:** Top 10 targets of the compound-target-pathway-disease network.

No.	Target	Degree
1	EGFR	30
2	CA2	29
3	CA4	29
4	AKT1	28
5	AKR1B1	27
6	PIK3R1	26
7	MAPK1	23
8	PIK3CB	22
9	IGF1R	22
10	MET	22

**Table 5:** Top 10 pathways of the compound-target-pathway-disease network.

No.	Pathway	Degree
hsa05200	Pathways in cancer	91
hsa04151	PI3K-Akt signaling pathway	58
hsa04010	MAPK signaling pathway	52
hsa05417	Lipid and atherosclerosis	49
hsa05205	Proteoglycans in cancer	43
hsa05208	Chemical carcinogenesis—reactive oxygen species	43
hsa04014	Ras signaling pathway	43
hsa05167	Kaposi sarcoma-associated herpesvirus infection	41
hsa05207	Chemical carcinogenesis—receptor activation	41
hsa05161	Hepatitis B	38

## Data Availability

The data that support the findings of this study are available from the corresponding authors upon reasonable request.

## Supporting Information

The name, molecular formula, batch number, and company of reference compounds are shown in Table S1. The top 20 GO entries and KEGG pathways obtained from GO and KEGG enrichment analysis are shown in Tables S2-S3.
