# Supplementary material for: Characterization of the Active Ingredients and Prediction of the Potential Anticolitis Mechanism of the Feng-Liao-Chang-Wei-Kang Capsule via Mass Spectrometry and Network Pharmacology
Source: J Anal Methods Chem. 2025 May 5;2025:2948965. doi: 10.1155/jamc/2948965 (PMC12069849; doi:10.1155/jamc/2948965)
Supplement: Supporting Information — Additional supporting information can be found online in the Supporting Information section. [file 2948965.f1.docx]

Characterization of the Active Ingredients and Prediction of the Potential Anti-colitis Mechanism of Feng-Liao-Chang-Wei-Kang Capsule via Mass Spectrometry and Network Pharmacology

Tingting Liu^1&^, Zhijiang He^2&^, Witiao Lv^3&^, Liyun Deng^3^, Xizhe Sun^1*^, Yanfei Chen^1*^

^1^School of Hainan Provincial Drug Safety Evaluation Research Center, Hainan Medical University, Haikou, China

^2^Department of Orthopedics, Hainan Provincial Corps Hospital of Chinese People’s Armed Police Force, Haikou, China

^3^School of Pharmacy, Hainan Medical University, Haikou, China

* Correspondence:
Yanfei Chen

E-mail: hy0308024@hainmc.edu.cn

Xizhe Sun

E-mail: hy0308028@hainmc.edu.cn

*These authors contributed equally to this work and should be considered co-correspondence authors.

& These authors contributed equally to this work and should be considered co-first

authors.

TABLE S1 The information of reference compound

| No. | Compound | Molecular formula | Batch number | Company |
| --- | --- | --- | --- | --- |
| 1 | Mannosamine | C_6_H_13_NO_5_ | Lsmls-lot-150-88 | Sigma-Aldrich (St. Louis, MO, USA) |
| 2 | Iminodiacetic acid | C_4_H_7_NO_4_ | HY-W012683 | Chemexpress Co., Ltd. (Shanghai, China) |
| 3 | L-Histidine | C_6_H_9_N_3_O_2_ | HY-N0832 | Chemexpress Co., Ltd. (Shanghai, China) |
| 4 | L-Arginine | C_6_H_14_N_4_O_2_ | HY-N0455 | Chemexpress Co., Ltd. (Shanghai, China) |
| 5 | 3-Pyridineacetic acid | C_7_H_7_NO_2_ | HY-W015806 | Chemexpress Co., Ltd. (Shanghai, China) |
| 6 | Melezitose | C_18_H_32_O_16_ | HY-N2340 | Chemexpress Co., Ltd. (Shanghai, China) |
| 7 | Turanose | C_12_H_22_O_11_ | HY-113334 | Chemexpress Co., Ltd. (Shanghai, China) |
| 8 | Gluconic acid | C_6_H_12_O_7_ | HY-Y0569 | Chemexpress Co., Ltd. (Shanghai, China) |
| 9 | Gentianose | C_18_H_32_O_16_ | HY-N8305 | Chemexpress Co., Ltd. (Shanghai, China) |
| 10 | Threonic acid | C_4_H_8_O_5_ | HY-W009208 | Chemexpress Co., Ltd. (Shanghai, China) |
| 11 | Stachydrine | C_7_H_13_NO_2_ | HY-N0298 | Chemexpress Co., Ltd. (Shanghai, China) |
| 12 | FAPy-adenine | C_5_H_7_N_5_O | HY-113303 | Chemexpress Co., Ltd. (Shanghai, China) |
| 13 | Glucose | C_6_H_12_O_6_ | HY-128417 | Chemexpress Co., Ltd. (Shanghai, China) |
| 14 | Glycolaldehyde dimer | C_4_H_8_O_4_ | HY-W004661 | Chemexpress Co., Ltd. (Shanghai, China) |
| 15 | Citric acid | C_6_H_8_O_7_ | HY-N1428 | Chemexpress Co., Ltd. (Shanghai, China) |
| 16 | Malic acid | C_4_H_6_O_5_ | HY-Y1069 | Chemexpress Co., Ltd. (Shanghai, China) |
| 17 | Uridine | C_9_H_12_N_2_O_6_ | HY-B1449 | Chemexpress Co., Ltd. (Shanghai, China) |
| 18 | 6-Methylnicotinamide | C_7_H_8_N_2_O | HY-W015815 | Chemexpress Co., Ltd. (Shanghai, China) |
| 19 | L-Isoleucine | C_6_H_13_NO_2_ | HY-N0771 | Chemexpress Co., Ltd. (Shanghai, China) |
| 20 | Adenosine | C_10_H_13_N_5_O_4_ | HY-B0228 | Chemexpress Co., Ltd. (Shanghai, China) |
| 21 | Methylmalonic acid | C_4_H_6_O_4_ | HY-103395 | Chemexpress Co., Ltd. (Shanghai, China) |
| 22 | Gallic acid | C_7_H_6_O_5_ | HY-N0523 | Chemexpress Co., Ltd. (Shanghai, China) |
| 23 | Protocatechuic acid | C_7_H_6_O_4_ | HY-N0294 | Chemexpress Co., Ltd. (Shanghai, China) |
| 24 | Geniposidic acid | C_16_H_22_O_10_ | HY-N0010 | Chemexpress Co., Ltd. (Shanghai, China) |
| 25 | Mussaenosidic acid | C_16_H_24_O_10_ | B28673-5mg | Shanghai yuanye Bio-Technology Co., Ltd (Shanghai, China) |
| 26 | 8-Epi-Loganic acid-6'-O-beta-D-glucoside | C_22_H_34_O_15_ | DB0165-0010 | Chengdu Lemeitian Pharmaceutical Technology Co., Ltd. (Chengdu, China) |
| 27 | 4-O-beta-Glucopyranosyl-cis-coumaric acid | C_15_H_18_O_8_ | DX0066 | Chengdu Lemeitian Pharmaceutical Technology Co., Ltd. (Chengdu, China) |
| 28 | 5'-Methylthioadenosine | C_11_H_15_N_5_O_3_S | HY-16938 | Chemexpress Co., Ltd. (Shanghai, China) |
| 29 | Asperulosidic Acid | C_18_H_24_O_12_ | HY-N6246 | Chemexpress Co., Ltd. (Shanghai, China) |
| 30 | Kaempferol 3-sophoroside-7-glucoside | C_33_H_40_O_21_ | DS0115-0005 | Chengdu Lemeitian Pharmaceutical Technology Co., Ltd. (Chengdu, China) |
| 31 | Trans-ferulic acid-4-beta-glucoside | C_16_H_20_O_9_ | Y69061-50mg | Shanghai yuanye Bio-Technology Co., Ltd. (Shanghai, China) |
| 32 | Crenulatin | C_11_H_20_O_6_ | DD0302 | Chengdu Lemeitian Pharmaceutical Technology Co., Ltd. (Chengdu, China) |
| 33 | Syringin | C_17_H_24_O_9_ | HY-N0824 | Chemexpress Co., Ltd. (Shanghai, China) |
| 34 | Catechin | C_15_H_14_O_6_ | HY-N0898 | Chemexpress Co., Ltd. (Shanghai, China) |
| 35 | Asperuloside | C_18_H_22_O_11_ | HY-N1382 | Chemexpress Co., Ltd. (Shanghai, China) |
| 36 | Oxyresveratrol 2-O-beta-D-glucopyranoside | C_20_H_22_O_9_ | HY-N3516 | Chemexpress Co., Ltd. (Shanghai, China) |
| 37 | Afzelechin | C_15_H_14_O_5_ | B30001-5mg | Shanghai yuanye Bio-Technology Co., Ltd. (Shanghai, China) |
| 38 | Roseoside | C_19_H_30_O_8_ | STC6030205 | Shanghai Standard Biotech Co., Ltd. (Shanghai, China) |
| 39 | Epicatechin | C_15_H_14_O_6_ | HY-N0001 | Chemexpress Co., Ltd. (Shanghai, China) |
| 40 | Manghaslin | C_33_H_40_O_20_ | DH0313-0010 | Chengdu Lemeitian Pharmaceutical Technology Co., Ltd. (Chengdu, China) |
| 41 | Isovanillic acid | C_8_H_8_O_4_ | HY-N6864 | Chemexpress Co., Ltd. (Shanghai, China) |
| 42 | Megastigm-7-ene-3,5,6,9-tetraol | C_13_H_24_O_4_ | B22007-10mg | Shanghai yuanye Bio-Technology Co., Ltd. (Shanghai, China) |
| 43 | Paeonolide | C_20_H_28_O_12_ | HY-N2156 | Chemexpress Co., Ltd. (Shanghai, China) |
| 44 | Butin-7-O-β-D-glucopyranoside | C_21_H_22_O_10_ | DZ0163-0005 | Chengdu Lemeitian Pharmaceutical Technology Co., Ltd. (Chengdu, China) |
| 45 | Mauritianin | C_33_H_40_O_19_ | HY-N5038 | Chemexpress Co., Ltd. (Shanghai, China) |
| 46 | Lyoniresinol 9'-O-glucoside | C_28_H_38_O_13_ | DN0103-0005 | Chengdu Lemeitian Pharmaceutical Technology Co., Ltd. (Chengdu, China) |
| 47 | Quercetin 3-o-neohesperidoside | C_27_H_30_O_16_ | DH0164-0010 | Chengdu Lemeitian Pharmaceutical Technology Co., Ltd. (Chengdu, China) |
| 48 | Myricetin 3-O-rutinoside | C_27_H_30_O_17_ | DY0717 | Chengdu Lemeitian Pharmaceutical Technology Co., Ltd. (Chengdu, China) |
| 49 | Rutin | C_27_H_30_O_16_ | HY-N0148 | Chemexpress Co., Ltd. (Shanghai, China) |
| 50 | Quercetin-3-O-glucuronide | C_21_H_18_O_13_ | HY-13930 | Chemexpress Co., Ltd. (Shanghai, China) |
| 51 | Hyperoside | C_21_H_20_O_12_ | HY-N0419 | Chemexpress Co., Ltd. (Shanghai, China) |
| 52 | Myricitrin | C_21_H_20_O_12_ | HY-N0152 | Chemexpress Co., Ltd. (Shanghai, China) |
| 53 | Apigenin 5-O-glucoside | C_21_H_20_O_10_ | DA0115 | Chengdu Lemeitian Pharmaceutical Technology Co., Ltd. (Chengdu, China) |
| 54 | Quercetin 3-o-(6''-galloyl)-beta-d-glucopyranoside | C_28_H_24_O_16_ | STC0420105 | Shanghai Standard Biotech Co., Ltd. (Shanghai, China) |
| 55 | Suberic acid | C_8_H_14_O_4_ | HY-W015300 | Chemexpress Co., Ltd. (Shanghai, China) |
| 56 | Ellagic acid | C_14_H_6_O_8_ | HY-B0183 | Chemexpress Co., Ltd. (Shanghai, China) |
| 57 | m-Anisaldehyde | C_8_H_8_O_2_ | HY-W007346 | Chemexpress Co., Ltd. (Shanghai, China) |
| 58 | Kaempferol-3-O-rutinoside | C_27_H_30_O_15_ | HY-N0208 | Chemexpress Co., Ltd. (Shanghai, China) |
| 59 | Kaempferol 3-O-robinobioside | C_27_H_30_O_15_ | DB0309-0010 | Chengdu Lemeitian Pharmaceutical Technology Co., Ltd. (Chengdu, China) |
| 60 | L-3-Phenyllactic acid | C_9_H_10_O_3_ | HY-30220 | Chemexpress Co., Ltd. (Shanghai, China) |
| 61 | Trifolin | C_21_H_20_O_11_ | DS0235-0010 | Chengdu Lemeitian Pharmaceutical Technology Co., Ltd. (Chengdu, China) |
| 62 | Quercitrin | C_21_H_20_O_11_ | HY-N0015 | Chemexpress Co., Ltd. (Shanghai, China) |
| 63 | Vitexin | C_21_H_20_O_10_ | HY-N0013 | Chemexpress Co., Ltd. (Shanghai, China) |
| 64 | Sophoricoside | C_21_H_20_O_10_ | HY-N0423 | Chemexpress Co., Ltd. (Shanghai, China) |
| 65 | Aloeresin D | C_29_H_32_O_11_ | HY-N2215 | Chemexpress Co., Ltd. (Shanghai, China) |
| 66 | Okanin | C_15_H_12_O_6_ | HY-N6673 | Chemexpress Co., Ltd. (Shanghai, China) |
| 67 | Steppogenin | C_15_H_12_O_6_ | DC0242-0005 | Chengdu Lemeitian Pharmaceutical Technology Co., Ltd. (Chengdu, China) |
| 68 | Chrysophanein | C_21_H_20_O_9_ | HY-N4151 | Chemexpress Co., Ltd. (Shanghai, China) |
| 69 | Sebacic acid | C_10_H_18_O_4_ | HY-W014787 | Chemexpress Co., Ltd. (Shanghai, China) |
| 70 | Luteolin | C_15_H_10_O_6_ | HY-N0162 | Chemexpress Co., Ltd. (Shanghai, China) |
| 71 | Quercetin | C_15_H_10_O_7_ | HY-18085 | Chemexpress Co., Ltd. (Shanghai, China) |
| 72 | Morin | C_15_H_10_O_7_ | HY-N0621 | Chemexpress Co., Ltd. (Shanghai, China) |
| 73 | Syringaresinol | C_22_H_26_O_8_ | HY-126066 | Chemexpress Co., Ltd. (Shanghai, China) |
| 74 | 3-O-Methylquercetin | C_16_H_12_O_7_ | HY-N1860 | Chemexpress Co., Ltd. (Shanghai, China) |
| 75 | Naringenin chalcone | C_15_H_12_O_5_ | HY-N3007 | Chemexpress Co., Ltd. (Shanghai, China) |
| 76 | Apigenin | C_15_H_10_O_5_ | HY-N1201 | Chemexpress Co., Ltd. (Shanghai, China) |
| 77 | Kaempferol | C_15_H_10_O_6_ | HY-14590 | Chemexpress Co., Ltd. (Shanghai, China) |
| 78 | Alpinetin | C_16_H_14_O_4_ | HY-N0625A | Chemexpress Co., Ltd. (Shanghai, China) |
| 79 | 2-Methoxycinnamaldehyde | C_10_H_10_O_2_ | HY-W046353 | Chemexpress Co., Ltd. (Shanghai, China) |
| 80 | Dodecanedioic acid | C_12_H_22_O_4_ | HY-W012241 | Chemexpress Co., Ltd. (Shanghai, China) |
| 81 | 3-Hydroxycapric acid | C_10_H_20_O_3_ | HY-113057 | Chemexpress Co., Ltd. (Shanghai, China) |
| 82 | p-Hydroxyphenethyl trans-ferulate | C_18_H_18_O_5_ | DH0304 | Chengdu Lemeitian Pharmaceutical Technology Co., Ltd. (Chengdu, China) |
| 83 | 4',5-Dihydroxyflavone | C_15_H_10_O_4_ | HY-N1881 | Chemexpress Co., Ltd. (Shanghai, China) |
| 84 | Dihydroartemisinin | C_15_H_24_O_5_ | HY-N0176 | Chemexpress Co., Ltd. (Shanghai, China) |
| 85 | Acacetin | C_16_H_12_O_5_ | HY-N0451 | Chemexpress Co., Ltd. (Shanghai, China) |
| 86 | Desmethoxyyangonin | C_14_H_12_O_3_ | HY-N0918 | Chemexpress Co., Ltd. (Shanghai, China) |
| 87 | L-Borneol | C_10_H_18_O | JOT-10228 | Chengdu Pufei De Biotech Co., Ltd. (Chengdu, China) |
| 88 | 15-Hydroxydehydroabietic acid | C_20_H_28_O_3_ | DQ0045 | Chengdu Lemeitian Pharmaceutical Technology Co., Ltd. (Chengdu, China) |
| 89 | Cardamonin | C_16_H_14_O_4_ | HY-N0279 | Chemexpress Co., Ltd. (Shanghai, China) |
| 90 | 3-Hydroxydodecanoic acid | C_12_H_24_O_3_ | HY-113107 | Chemexpress Co., Ltd. (Shanghai, China) |
| 91 | Hexadecanedioic acid | C_16_H_30_O_4_ | HY-W018161 | Chemexpress Co., Ltd. (Shanghai, China) |
| 92 | Pelargonic acid | C_9_H_18_O_2_ | HY-N7057 | Chemexpress Co., Ltd. (Shanghai, China) |
| 93 | Pinostrobin | C_16_H_14_O_4_ | HY-N2127 | Chemexpress Co., Ltd. (Shanghai, China) |
| 94 | Pellitorine | C_14_H_25_NO | HY-N3097 | Chemexpress Co., Ltd. (Shanghai, China) |
| 95 | Ricinoleic acid | C_18_H_34_O_3_ | famls-lot-205-47 | Sigma-Aldrich (St. Louis, MO, USA) |
| 96 | Lupenone | C_30_H_48_O | HY-N2590 | Chemexpress Co., Ltd. (Shanghai, China) |
| 97 | Octadecanedioic acid | C_18_H_34_O_4_ | HY-W005178 | Chemexpress Co., Ltd. (Shanghai, China) |
| 98 | 2-Hydroxytetradecanoic acid | C_14_H_28_O_3_ | famls-lot-205-47 | Sigma-Aldrich (St. Louis, MO, USA) |
| 99 | Oleamide | C_18_H_35_NO | HY-N2327 | Chemexpress Co., Ltd. (Shanghai, China) |

TABLE S2 Top 20 GO entries

| GO entries | No. | Term | Count | *P* value |
| --- | --- | --- | --- | --- |
| BP | 1 | protein phosphorylation | 76 | 3.76E-56 |
|  | 2 | phosphorylation | 88 | 3.09E-52 |
|  | 3 | protein autophosphorylation | 37 | 2.77E-30 |
|  | 4 | response to xenobiotic stimulus | 42 | 2.83E-27 |
|  | 5 | negative regulation of apoptotic process | 55 | 3.42E-26 |
|  | 6 | inflammatory response | 50 | 1.11E-25 |
|  | 7 | peptidyl-tyrosine phosphorylation | 23 | 1.54E-25 |
|  | 8 | peptidyl-serine phosphorylation | 33 | 2.86E-24 |
|  | 9 | signal transduction | 75 | 6.29E-20 |
|  | 10 | positive regulation of cell proliferation | 47 | 1.28E-19 |
|  | 11 | transmembrane receptor protein tyrosine kinase signaling pathway | 25 | 2.48E-19 |
|  | 12 | response to lipopolysaccharide | 27 | 3.67E-19 |
|  | 13 | positive regulation of kinase activity | 19 | 2.41E-18 |
|  | 14 | positive regulation of gene expression | 45 | 4.89E-18 |
|  | 15 | positive regulation of protein kinase B signaling | 29 | 6.23E-18 |
|  | 16 | positive regulation of cell migration | 33 | 7.95E-18 |
|  | 17 | multicellular organism development | 21 | 9.07E-18 |
|  | 18 | positive regulation of MAPK cascade | 27 | 4.17E-17 |
|  | 19 | positive regulation of ERK1 and ERK2 cascade | 30 | 1.01E-16 |
|  | 20 | xenobiotic metabolic process | 23 | 1.54E-16 |
| CC | 1 | plasma membrane | 190 | 9.97E-29 |
|  | 2 | receptor complex | 35 | 2.04E-23 |
|  | 3 | cytosol | 171 | 2.09E-18 |
|  | 4 | membrane raft | 28 | 2.54E-16 |
|  | 5 | cytoplasm | 157 | 3.68E-12 |
|  | 6 | perinuclear region of cytoplasm | 41 | 1.51E-10 |
|  | 7 | extrinsic component of cytoplasmic side of plasma membrane | 11 | 4.24E-10 |
|  | 8 | cell surface | 37 | 4.53E-10 |
|  | 9 | nucleoplasm | 117 | 5.40E-10 |
|  | 10 | macromolecular complex | 38 | 8.81E-10 |
|  | 11 | external side of plasma membrane | 27 | 9.17E-09 |
|  | 12 | axon | 25 | 1.05E-08 |
|  | 13 | membrane | 145 | 1.10E-08 |
|  | 14 | focal adhesion | 27 | 2.56E-08 |
|  | 15 | dendrite | 27 | 4.69E-08 |
|  | 16 | extracellular region | 72 | 5.08E-08 |
|  | 17 | caveola | 12 | 5.62E-08 |
|  | 18 | extracellular exosome | 72 | 1.75E-07 |
|  | 19 | lysosome | 21 | 1.90E-07 |
|  | 20 | glutamatergic synapse | 24 | 8.32E-07 |
| MF | 1 | ATP binding | 115 | 5.78E-40 |
|  | 2 | protein kinase activity | 61 | 5.63E-38 |
|  | 3 | protein tyrosine kinase activity | 37 | 8.94E-35 |
|  | 4 | protein serine/threonine kinase activity | 51 | 9.62E-28 |
|  | 5 | RNA polymerase II transcription factor activity, ligand-activated sequence-specific DNA binding | 25 | 1.09E-27 |
|  | 6 | enzyme binding | 49 | 1.60E-26 |
|  | 7 | transmembrane receptor protein tyrosine kinase activity | 20 | 2.38E-22 |
|  | 8 | kinase activity | 35 | 1.57E-20 |
|  | 9 | non-membrane spanning protein tyrosine kinase activity | 17 | 8.37E-17 |
|  | 10 | zinc ion binding | 57 | 8.13E-16 |
|  | 11 | identical protein binding | 82 | 4.02E-15 |
|  | 12 | receptor binding | 35 | 3.48E-14 |
|  | 13 | heme binding | 21 | 1.06E-11 |
|  | 14 | endopeptidase activity | 15 | 6.24E-11 |
|  | 15 | peptidase activity | 16 | 3.67E-10 |
|  | 16 | protein homodimerization activity | 42 | 5.11E-10 |
|  | 17 | transcription coactivator binding | 12 | 9.57E-10 |
|  | 18 | protein phosphatase binding | 15 | 9.84E-10 |
|  | 19 | protein binding | 287 | 1.47E-09 |
|  | 20 | iron ion binding | 18 | 1.56E-09 |

TABLE S3 Top 20 KEGG pathways

| No. | Term | Count | *P* value |
| --- | --- | --- | --- |
| 1 | Pathways in cancer | 90 | 3.88E-36 |
| 2 | Lipid and atherosclerosis | 48 | 6.06E-24 |
| 3 | Prostate cancer | 34 | 1.25E-23 |
| 4 | EGFR tyrosine kinase inhibitor resistance | 31 | 3.41E-23 |
| 5 | AGE-RAGE signaling pathway in diabetic complications | 32 | 6.96E-21 |
| 6 | PI3K-Akt signaling pathway | 57 | 7.28E-21 |
| 7 | Non-small cell lung cancer | 28 | 8.28E-21 |
| 8 | PD-L1 expression and PD-1 checkpoint pathway in cancer | 30 | 2.76E-20 |
| 9 | Endocrine resistance | 31 | 4.56E-20 |
| 10 | MAPK signaling pathway | 51 | 7.08E-20 |
| 11 | HIF-1 signaling pathway | 32 | 1.19E-19 |
| 12 | Proteoglycans in cancer | 42 | 1.88E-19 |
| 13 | Pancreatic cancer | 27 | 6.60E-19 |
| 14 | Hepatitis B | 37 | 9.18E-19 |
| 15 | Kaposi sarcoma-associated herpesvirus infection | 40 | 1.26E-18 |
| 16 | Chemical carcinogenesis - reactive oxygen species | 42 | 4.68E-18 |
| 17 | Neurotrophin signaling pathway | 31 | 1.96E-17 |
| 18 | Chemical carcinogenesis - receptor activation | 40 | 3.16E-17 |
| 19 | T cell receptor signaling pathway | 31 | 3.23E-17 |
| 20 | Ras signaling pathway | 42 | 3.87E-17 |
